# Cancer incidence and mortality and risk factors in member countries of the " Belt and Road " initiative

**DOI:** 10.1186/s12885-022-09657-3

**Published:** 2022-05-25

**Authors:** Baohua Wang, Fengdie He, Yanan Hu, Qiutong Wang, Dan Wang, Yuting Sha, Jing Wu

**Affiliations:** 1 National Center for Chronic and Noncommunicable Disease Control and Prevention, Chinese Center for Disease Control and Prevention, Beijing, China (Mailing address: 27 Nanwei Road, Xicheng District, Beijing, 100050 China; 2 School of public health, China Medical University, Shenyang, China (Mailing address: 77 Puhe Road, Shenbei New District, Shenyang, 110000 China

**Keywords:** “B&R”, Cancer incidence, Cancer mortality, Risk factors, Cancer prevention strategies

## Abstract

**Background:**

At present, “Belt and Road” (“B&R”) member states (accounting for about 61.78% of the world’s population) face different types of cancer threats to varying degrees. We analyzed the incidence and mortality and risk factors of cancer in the member countries of the “B&R” to explore the basis of health and medical cooperation between countries and provide a foundation for formulating cancer prevention and control policies for building a healthy "B&R."

**Methods:**

Data were derived from the Global Cancer Observatory and Cancer Country Profiles in 2020. Incidence and mortality were age-standardized rates (ASRs). Population attributable fractions (PAFs) was applied to measure risk factors of cancers in the “B&R” countries. The mortality­to­incidence ratio (MIR) was calculated by dividing the mortality rate by the incidence rate.

**Results:**

A total of 26 cancers were included in the study. Lung, breast, colorectal, stomach, liver, prostate, cervical, esophageal, thyroid, and uterine cancers were the most common and highest in age-standardized mortality in the “B&R” countries. For men, Hungary had the highest cancer age-standardized incidence and mortality (ASR, 289.3 per 100,000 and ASR, 235.7 per 100,000, respectively), followed by Latvia (ASR, 288.6 per 100,000 and ASR, 196.5 per 100,000, respectively). In females, the highest incidence rates were estimated in Greece (ASR, 238.7 per 100,000), and the highest mortality rate was Brunei (ASR, 192.3 per 100,000). All countries were in the middle or high HDI range, with about half (46.88%) of countries achieving high HDI, mostly in Central and Eastern Europe (13 countries) and West Asia (10 countries). The United Arab Emirates had the highest MIR in male and female (1.59 vs 2.19). Tobacco products, infectious factors, and ultraviolet rays were the three main cancer risk factors in the “B&R” countries.

**Conclusion:**

The overall burden of cancer in the countries along the “B&R” remains substantial, while the corresponding cancer prevention and control policies need to be improved. Strengthening health cooperation among member countries will contribute to a joint response to the risks and challenges posed by cancer.

**Supplementary Information:**

The online version contains supplementary material available at 10.1186/s12885-022-09657-3.

## Introduction

More than 2,000 years ago, people on the Eurasian continent explored many trade and cultural exchange routes that connected the major civilizations of Asia, Europe, and Africa, and later generations collectively referred to these routes as the "Silk Road" [1]. During Chinese President Xi Jinping’s visits to Central and Southeast Asian countries in September and October 2013, he proposed jointly building the "Silk Road Economic Belt" and the "21st Century Maritime Silk Road." The "B&R" achieves the common development and common prosperity of all member states by building a community of interest, destiny, and responsibility in all aspects of political mutual trust, economic integration, and cultural inclusiveness.

In recent years, with the development of society and the economy, the disease burden in low–human development index (HDI) and medium-HDI countries has shifted from being dominated by communicable, maternal, neonatal, and nutritional diseases (CMNNDS) to being dominated by non-communicable diseases (NCDs) and injuries [2]. With the improvement of clinical diagnosis and treatment, cancer is among the ranks of chronic non-infectious diseases and is one of the main components of the NCDs burden. Cancer is the second leading cause of deaths after cardiovascular disease in the world, accounting for about 10.1 million deaths (17.83% of total deaths) in 2019 [3]. As health is a core issue of human development, China's "B&R" initiative provides a great opportunity for cooperation and collective action among multiple countries. In 2016, working together to build a healthy Silk Road, taking health as one of the important contents of the “B&R” construction was proposed by Xi Jinping. The development of health undertakings is directly related to the national and regional SDG and was the driving force for the construction of the "B&R" [4]. At present, member states face different types of cancers threats to varying degrees. Understanding the status quo and risk factors of cancer in the “B&R” member states is an important foundation for health cooperation between countries. Therefore, based on global data of cancer and cancer country profiles released by the World Health Organization in 2020, we analyzed the incidence, mortality, and risk factors of cancer in the member countries of the “B&R” to provide a basis for formulating cancer prevention and control policies for building a healthy "B&R."

## Materials and methods

### Data source

We used data from the Global Cancer Observatory (gco.iarc.fr) [5] and Cancer Country Profiles (https://www.who.int/countries) [6] in 2020. Global Cancer Observatory, an online data set that provides a comprehensive assessment of the Global Cancer burden in 2020 based on GLOBOCAN estimates of incidence, mortality for 36 cancer types, grouped by sex and age, in 185 countries or territories in 2020. The methods used in compiling the estimates in GLOBOCAN were country-specific, and the quality of the national estimates depended on the coverage, accuracy, and timeliness of the recorded incidence and mortality data in each country. More details are described in other literature [7].

"B&R" initiative refers to the cooperative organization of politics, economics, culture, and other related fields, and includes 66 member countries. Apart from Cyprus, the Czech Republic, Greece, Hungary, Israel, and Singapore, the remaining 60 countries are all developing countries: (1) East Asia: China, Mongolia; (2) The association of southeast Asian nations (ASEAN): Singapore, Malaysia, Indonesia, Myanmar, Thailand, Laos, Cambodia, Vietnam, Brunei, and the Philippines; (3) West Asia: Iran, Iraq, Turkey, Syria, Jordan, Lebanon, Israel, Palestine, Saudi Arabia, Yemen, Oman, United Arab Emirates, Qatar, Kuwait, Bahrain, Greece, Cyprus, and Egypt's Sinai Peninsula; (4) South Asia: India, Pakistan, Bangladesh, Afghanistan, Sri Lanka, the Maldives, Nepal, and Bhutan; (5) Central Asia: Kazakhstan, Uzbekistan, Turkmenistan, Tajikistan, and Kyrgyzstan; (6) The Commonwealth of Independent States (CIS): Russia, Ukraine, Belarus, Georgia, Azerbaijan, Armenia, and the Republic of Moldova; (7) Central and Eastern Europe (CEE): Poland, Lithuania, Estonia, Latvia, Czech Republic, Slovakia, Hungary, Slovenia, Croatia, Bosnia and Herzegovina, Montenegro, Serbia, Albania, Romania, Bulgaria, and Macedonia. The division of the “B&R” countries is based on the World Bank's division of global regions and international political and economic organizations. There is a lack of cancer-related data in Palestine and Yemen in this database. Therefore, we collected and analyzed cancer data in the other 64 member states.

### Index definition

In this study, we used incidence, mortality, and age-standardized rates (ASRs) to analyze and compare cancer incidence and death in "B&R" countries. Incidence, generated by population-based cancer registries (PBCRs), is the number of new cases occurring in a specified period and geographic region, and conveyed either as an absolute number of cases per annum or as a rate per 100,000 people per year. Mortality, similarly, is used to measure the number of deaths per unit of time (overall or attributable to specific factors) in a population of a given size. It is usually expressed in units per 100,000 people per year. ASRs per 100,000 people-years are corrected by the direct method and the world standard population to allow for differences in the age structure of a population. The methods used to estimate the global incidence and mortality in 2020 (for all ages) can be found in other literature [8, 9]. We calculated the mortality­to­incidence ratio (MIR) by dividing the mortality rate by the incidence rate [10, 11]. The population attributable fractions (PAFs) refer to the proportion of the incidence or death of a disease attributable to a certain exposure factor in the total incidence or death of a disease in the population, that is, the proportion of the incidence or death of related diseases that can be reduced in the population after the elimination of exposure factors. The specific calculation method is detailed in other literature [12].

### Data analysis

We conducted a descriptive analysis and comparison of the incidence and deaths due to cancers in the “B&R” countries, as well as a visual presentation. Microsoft Excel 2019 was used for data visualization.

## Results

### Age-standardized incidence and mortality of cancers in global and geographical distribution of the “B&R” countries

**Fig 1:**
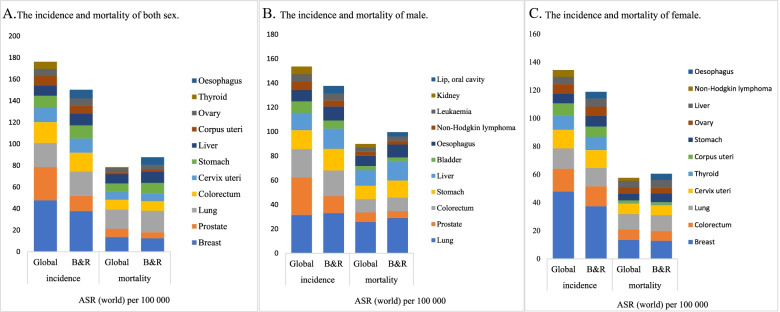
Comparison of estimated 2020 age-standardized rates (world) between Global and “One Belt &One Road” countries, all ages

A total of 26 cancers were included in the study. Breast, lung, colorectum, prostate, stomach, liver, cervix uteri, esophagus, thyroid, and bladder cancer ranked amongst the top 10 new cancer in 2020, and lung, colorectum, liver, stomach, breast, oesophagus, pancreas, prostate, cervix uteri, and leukaemia cancer ranked among the top 10 cancer in terms of deaths, with a total of 12,176,526 new cases and 7,054,094 deaths worldwide, respectively (Table 1). Along the “B&R” member countries, the top 10 cancers with the highest incidence rate were lung, breast, colorectum, stomach, liver, esophagus, prostate, cervix uteri, thyroid, and corpus uteri cancers. The top 10 cancers with the highest mortality rate were lung, liver, stomach, colorectum, esophagus, breast, cervix uteri, prostate, pancreas, and ovary cancers. The member countries of the “B&R” had lower cancer incidence and higher cancer mortality compared to the world (Fig. 1A). The cancer with the highest incidence and mortality ASRs was lung cancer in the world and the “B&R” member countries in male, while breast cancer in female (Fig. 1B, C).

**Table 1:** New Cases and Deaths of main cancers for global and geographical location of the “B&R” countries Combined in 2020[n]

**Caner Site**	**World**		**East Asia**		**ASEAN**		**West Asia**		**South Asia**		**Central Asia**		**CIS**		**CEE**		
	**New cases**	**Deaths**	**New cases**	**Deaths**	**New cases**	**Deaths**	**New cases**	**Deaths**	**New cases**	**Deaths**	**New cases**	**Deaths**	**New cases**	**Deaths**	**New cases**		**Deaths**
**Breast**	2261419	684996	416559	117236	158820	58622	99116	33153	226537	115464	11377	4701	104903	34470	77907	25376	
**Lung**	2206771	1796144	816048	715119	123221	109442	77575	68712	99734	91007	8717	7089	89929	76256	87552	77016	
**Colorectum**	1931590	935173	555637	286262	106919	57022	61339	31821	84176	48909	6869	4077	110426	59948	86171	46225	
**Prostate**	1414259	375304	115462	51111	41048	15925	48716	16279	43223	21071	2288	1172	65160	21066	64025	19731	
**Stomach**	1089103	768793	479368	374422	32012	26596	34358	29065	78719	69390	8992	6985	52187	39424	17842	14322	
**Liver**	905677	830180	412274	393212	99210	95617	39752	37598	28123	27307	3765	3585	8606	8199	5772	5334	
**Cervix uteri**	604127	341831	110075	59242	68249	38319	1487	694	142127	88663	4945	2678	22309	10820	13287	6729	
**Oesophagus**	604100	544076	324820	301508	10278	9704	-	-	98043	90947	3217	3053	886	865	120	103	
**Thyroid**	586202	43646	221093	9261	28475	3907	24919	2031	2058	325	-	-	17960	1242	7657	561	
**Bladder**	573278	212536	66242	29678	10810	5837	36575	14768	4632	2715	1454	655	20831	7928	30665	11997	
**Pancreas**	-	-	70500	67994	2449	2385	17020	16375	18	18	1449	1392	28433	27909	21937	20541	
**Melanoma of skin**	-	-	-	-	-	-	1167	194	-	-	-	-	2350	624	6979	1647	
**Corpus uteri**	-	-	81964	16607	24227	7238	15208	4097	20316	7699	2493	777	41911	9938	23653	5405	
**Ovary**	-	-	55423	37568	29734	19067	13036	8733	55407	38604	2357	1534	19413	11686	13024	8492	
**Non-Hodgkin lymphoma**	-	-	50155	29741	35466	20217	24762	12822	7929	4622	102	65	-	-	6528	2924	
**Leukemia**	-	-	-	-	34992	26537	27973	20005	59876	43969	848	715	9769	5976	2811	1717	
**Kidney**	-	-	118	42	2755	1338	3424	1440	386	268	1002	550	31322	12390	18075	7940	
**Brain, central nervous system**	-	-	34	30	514	446	13750	11603	1023	905	2150	1864	946	809	2175	1910	
**Hypopharynx**	-	-	-	-	3030	1760	-	-	28756	11529	-	-	-	-	-	-	
**Larynx**	-	-	-	-	1223	744	3986	1424	39161	23468	455	259	4068	2472	6264	3721	
**Lip, oral cavity**	-	-	-	-	8242	4780	-	-	171170	96257	744	374	13145	6489	6675	3321	
**Nasopharynx**	-	-	-	-	27190	18352	-	-	47	29	-	-	-	-	-	-	
**Hodgkin lymphoma**	-	-	-	-	-	-	123	31-	-	-	-	-	-	-	-	-	
**Gallbladder**	-	-	-	-	-	-	-	-	6353	4978	-	-	-	-	-	-	
**Oropharynx**	-	-	-	-	-	-	-	-	3266	2007	-	-	-	-	-	-	
**Total**	12176526	6532679	3775772	2489033	848864	523855	544286	310845	1201080	790151	63224	41525	644554	338511	499119	265012

### Distribution of cancer in each member country of the “B&R”

Table 2 shows the total number of the top 10 newly diagnosed cancer cases and deaths, as well as the incidence and mortality ASRs and MIR separately in each member country of the “B&R” in males and females. All countries were in the middle or high HDI range, with about half (46.88%) of countries achieving high HDI, mostly in Central and Eastern Europe (13 countries) and West Asia (10 countries). Among all the “B&R” member countries, the country with the highest incidence and mortality of cancers among males was Hungary (ASR, 289.3 per 100,000 and ASR, 235.7 per 100,000, respectively), followed by Latvia (ASR, 288.6 per 100,000 and ASR, 196.5 per 100,000, respectively), and the United Arab Emirates and Saudi Arabia, which enjoyed the lowest rates of incidence and mortality, respectively (ASR, 34.3 per 100,000 and ASR, 36.1 per 100,000, respectively). In females, the highest incidence rates were estimated in Greece (ASR, 238.7 per 100,000), followed by Mongolia, Turkey, Cyprus, Singapore, and Brunei. The country with the highest mortality rate was Brunei (ASR, 192.3 per 100,000), followed by Israel, Singapore, Cyprus, Greece, and Mongolia. Among the “B&R” member countries, the country with the highest cancer MIR among men was the United Arab Emirates (1.59), followed by Kuwait (1.17), and Turkey had the lowest MIR (0.45). Among women, the United Arab Emirates also had the highest MIR (2.19), followed by Afghanistan (1.64), and Belarus (0.37). See Table 2 for more details.

**Table 2:** HDI, Incidence, Mortality and MIR for “the Belt & Road” countries and Sex for the top ten cancers combined (Including Nonmelanoma Skin Cancer) in 2020

**Region**	**Country**	**population**	**The proportion in the world population (%)**	HDI*	**Incidence**				**Mortality**				**MIR**	
					**Males**		**Females**		**Males**		**Females**		**Males**	**Females**
					**Cases(n)**	**ASR** **(world)**	**Cases(n)**	**ASR** **(world)**	**Cases(n)**	**ASR** **(world)**	**Cases(n)**	**ASR** **(world)**		
East Asia														
China		1,402,112,000	18.09	0.761	2071503	110.9	1569685	152.5	1699192	57.6	915248	134.3	0.52	0.88
Mongolia		3,278,292	0.04	0.737	2731	208.3	2336	235.3	2346	126.1	1764	160.4	0.61	0.68
ASEAN														
Brunei		437,483	0.01	0.838	369	105.9	205	180	449	74	152	192.3	0.70	1.07
Cambodia		16,718,971	0.22	0.594	5,985	89	4,899	108	7,570	60.7	4,593	96.6	0.68	0.89
Indonesia		273,523,621	3.53	0.718	127,024	67.8	85,670	97.1	171,816	57.8	83,581	116.4	0.85	1.20
Laos		7,275,556	0.09	0.613	3,395	112.5	2,780	134.5	3,540	72.8	2,019	121.1	0.65	0.90
Malaysia		32,365,998	0.42	0.81	17,375	70.3	11,809	104.2	20,483	61.9	10,506	121.2	0.88	1.16
Myanmar		54,409,794	0.70	0.583	24,093	89.9	19,613	153.6	30,479	65.9	20,023	98.9	0.73	0.64
Philippines		109,581,085	1.41	0.718	51,138	93.4	36,435	127	71,068	66.7	34,434	137.5	0.71	1.08
Singapore		5,685,807	0.07	0.938	9,670	110.7	5,818	186	9,247	67.5	4,057	189.7	0.61	1.02
Thailand		69,799,978	0.90	0.777	70,395	94.1	52,769	128.7	76,657	57.6	42,656	134.3	0.61	1.04
Vietnam		97,338,583	1.26	0.704	79,763	89.9	62,988	153.6	68,348	57.6	38,848	134.3	0.64	0.87
West Asia														
Bahrain		1,701,583	0.02	0.852	418	50.7	228	82.7	519	46.6	202	103.4	0.92	1.25
Cyprus		1,207,361	0.02	0.887	2,217	95.81	1,128	216.9	1,791	57.6	740	185.4	0.60	0.85
Egypt		102,334,403	1.32	0.707	51,272	103.7	39,199	130.8	51,142	57.6	29,165	134.3	0.56	1.03
Greece		10,715,549	0.14	0.888	29,098	109.9	15,835	238.7	20,671	55.3	9,047	176.2	0.50	0.74
Iran		83,992,953	1.08	0.783	51,511	84	36,096	120.7	44,621	55.8	23,255	101.4	0.66	0.84
Iraq		40,222,503	0.52	0.674	9,721	67.2	6,856	91.2	14,805	56.7	7,312	107.2	0.84	1.18
Israel		9,216,900	0.12	0.919	11,236	89.9	5,034	153.6	11,375	56.1	4,352	190	0.62	1.24
Jordan		10,203,140	0.13	0.729	3,956	79.6	2,577	118.8	4,960	55.7	2,067	125.5	0.70	1.06
Kuwait		4,270,563	0.06	0.806	1,304	45.6	632	78	1,664	53.2	580	116.6	1.17	1.49
Lebanon		6,825,442	0.09	0.744	4,236	71.8	2,631	116.7	4,348	55.6	2,065	120.2	0.77	1.03
Oman		5,106,622	0.07	0.813	1,486	89.9	935	153.6	1,165	40.8	496	83.8	0.45	0.55
Qatar		2,881,060	0.04	0.848	632	50.1	320	79.6	457	51.8	166	103.4	1.03	1.30
Saudi Arabia		34,813,867	0.45	0.854	9,945	38.1	5,284	63.7	10,856	36.1	3,904	87.4	0.95	1.37
Syria		17,500,657	0.23	0.567	6,653	76.5	4,681	107.3	8,938	62.9	4,638	117.5	0.82	1.10
The united Arab emirates		34,813,867	0.45	0.89	1,530	34.3	688	61.3	2,186	54.7	639	134	1.59	2.19
Turkey		84,339,067	1.09	0.82	103,677	143.7	66,139	227.3	75,896	65	33,954	103.4	0.45	0.45
South Asia														
Afghanistan		38,928,341	0.50	0.511	6,776	61.4	5,611	73.2	8,437	59.82	5,676	119.9	0.97	1.64
Bangladesh		164,689,383	2.12	0.632	60,755	89.9	45,215	153.6	51,330	48.8	35,279	68.7	0.54	0.45
Bhutan		771,612	0.01	0.654	236	55.8	206	63.2	189	46.1	140	59.6	0.83	0.94
India		1,380,004,385	17.80	0.645	417,198	43.2	290,541	62	504,113	45.84	309,586	73.7	1.06	1.19
Maldives		540,542	0.01	0.74	194	68	136	94.1	211	51.1	88	115.4	0.75	1.23
Nepal		29,136,808	0.38	0.602	5,890	39.3	4,345	53.1	8,279	39.2	5,386	58.8	1.00	1.11
Pakistan		220,892,331	2.85	0.557	55,079	48.4	38,697	67.9	60,613	49.7	37,250	77.2	1.03	1.14
Sri Lanka		21,919,000	0.28	0.782	9,627	45.5	6,091	72.4	12,153	37.97	5,904	82.3	0.83	1.14
Central Asian														
Kazakhstan		18,754,440	0.24	0.825	12,181	89.9	8,751	153.6	13,802	56.9	7,406	114.4	0.63	0.74
Kyrgyzstan		6,591,600	0.09	0.697	2,481	89.9	2,020	153.6	2,927	57.5	1,752	94.1	0.64	0.61
Tajikistan		9,537,642	0.12	0.668	1,989	59.1	1,597	71.5	2,475	47.7	1,526	71.2	0.81	1.00
Turkmenistan		6,031,187	0.08	0.715	1,980	69.2	1,524	89.2	2,721	56.6	1,634	91	0.82	1.02
Uzbekistan		34,232,050	0.44	0.72	9,834	58	7,401	77.6	12,834	50.3	7,914	78.9	0.87	1.02
CIS														
Armenia		2,963,234	0.04	0.776	3,987	205.9	2,867	144.7	3,327	125.7	2,086	73.1	0.61	0.51
Azerbaijan		10,110,116	0.13	0.756	5,998	118.4	4,458	88.4	5,776	91	3,302	51.1	0.77	0.58
Belarus		9,398,861	0.12	0.823	16,723	244.7	9,296	134.6	15,288	167.8	6,031	49.6	0.69	0.37
Georgia		3,714,000	0.05	0.812	4,813	162.8	3,480	115.3	4,760	133.4	2,666	62.1	0.82	0.54
Moldova		2,617,820	0.03	0.75	5,658	209.7	3,883	143.9	4,718	133.6	2,590	66.9	0.64	0.46
Russia		144,104,080	1.86	0.824	216,520	210.1	130,042	124.6	235,235	163.2	105,157	58.3	0.78	0.47
Ukraine		44,134,693	0.57	0.779	58,905	181.9	35,480	108	62,846	148.5	27,173	52.2	0.82	0.48
CEE														
Albania		2,837,743	0.04	0.795	3,296	130.8	2,226	83.7	2,102	98.7	979	37.3	0.75	0.45
Bosnia and Herzegovina		3,280,815	0.04	0.78	6,169	196.1	4,475	137	4,837	149.9	2,834	73.5	0.76	0.54
Bulgaria		6,927,288	0.09	0.816	15,896	153.6	8,875	89.9	11,846	162	6,322	65.96	1.05	0.73
Croatia		4,047,200	0.05	0.851	10,628	251.8	6,383	134.78	8,667	192	4,201	66.75	0.76	0.50
Czechia		10,698,896	0.14	0.9	27,844	256.2	10,957	89.9	22,335	134.3	7,993	57.6	0.52	0.64
Estonia		1,331,057	0.02	0.892	3,406	287.2	1,662	127.1	2,781	180.3	1,346	55.6	0.63	0.44
Hungary		9,749,763	0.13	0.854	25,449	289.3	13,502	147.3	24,776	235.7	11,648	89.1	0.81	0.60
Latvia		1,901,548	0.02	0.866	4,868	288.6	2,346	131.8	4,380	196.5	2,008	67	0.68	0.51
Lithuania		2,794,700	0.04	0.882	6,609	230.6	3,177	99	6,045	134.3	2,553	57.6	0.58	0.58
Macedonia		2,083,380	0.03	0.774	3,448	200.4	2,223	123.1	2,702	157.1	1,287	66.8	0.78	0.54
Montenegro		621,718	0.01	0.829	1,109	210.4	772	137	1,036	134.3	526	57.6	0.64	0.42
Poland		37,950,802	0.49	0.88	78,597	221.9	49,900	137	76,615	195.6	38,215	77.2	0.88	0.56
Romania		19,286,123	0.25	0.828	40,773	230	24,545	133.7	33,599	174.1	16,596	67.81	0.76	0.51
Serbia		6,908,224	0.09	0.806	18,585	228.5	11,831	135.8	17,640	130.9	8,918	58.08	0.57	0.43
Slovakia		5,458,827	0.07	0.86	12,182	261.1	6,786	136.3	10,156	134.3	5,086	57.6	0.51	0.42
Slovenia		2,100,126	0.03	0.917	6,247	277.4	2,839	103.32	4,496	134.3	2,001	57.6	0.48	0.56

^*^Uniteed Nations Development Programme.Human Development Report 2020.http://hdr.undp.org/en/content/download-data.

Among the top 10 cancers in East Asian countries, the total incidence and mortality of cancer in Mongolia were ASR, 158.0 per 100,000 and ASR, 128.8 per 100,000, respectively, and both were larger in females than in males. Liver cancer had the highest cancer incidence and mortality. The total cancer incidence and mortality in China were 146.3 per 100,000 and 84.4 per 100,000, respectively, and lung cancer and breast cancer had the highest cancer incidence and mortality, respectively (supplementary Fig. S1).

Among ASEAN countries, Singapore had the highest cancer incidence (ASR, 149.4 per 100,000) and Vietnam had the highest cancer mortality (ASR, 58.9 per 100,000). The countries with the highest overall cancer incidence and mortality among males were Singapore and Laos (ASR, 132.9 per 100,000 and ASR, 80.4 per 100,000, respectively), while for females, the country was Brunei, with an overall cancer incidence and mortality of ASR, 137.4 per 100,000 and ASR, 52.9 per 100,000, respectively. Cancers of the colorectum, lung, prostate, and liver in men, and cancers of the breast, lung, colorectum, and cervix uteri in female, continue to be the most common fatal cancers (supplementary Fig. S2).

Among the member countries in West Asia, the highest cancer incidence rate was observed in Cyprus, Greece, and Israel, with a high cancer incidence of cancers of the breast, prostate, lung, and colorectum, while Turkey, Egypt, and Syria had the highest cancer mortality, and deaths associated with cancers of the lung, breast, liver, prostate, colorectum, and bladder deaths were the most numerous. Cancer incidence and mortality rates were highest among males in Greece (ASR, 238.7 per 100,000) and Turkey (ASR,143.7 per 100,000), with lung, prostate, bladder, and colorectal cancer being the main causes. Israel, Greece, and Cyprus had the highest cancer incidence rates of the breast, thyroid, corpus uteri, and colorectum in females. The top three countries with the highest cancer mortality were Cyprus, Egypt, and Iraq, where principal deaths of cancer were cancers of the breast, lung, colorectum, ovary, and cervix uteri; leukemia, brain, and central nervous system were all in the top five cancer death types in Iraq. See supplementary Fig. S3 for more details.

Large distribution differences existed in the incidence and mortality of cancers in South Asian member countries, with the total number of newly diagnosed cancer cases in Bhutan being much higher than that in other countries (ASR, 176.2 per 100,000). Bangladesh had some of the highest cancer incidence rates in males (ASR, 153.6 per 100,000), such as lung, prostate, and colorectal cancers. There was a high incidence of breast cancer, colorectal cancer, non-Hodgkin lymphoma, and esophageal cancers in females in Afghanistan (ASR, 119.9 per 100,000) and the Maldives (ASR, 115.4 per 100,000). On the contrary, deaths were more evenly distributed across countries, with the most deaths being from lung cancer and digestive system cancer in males and breast cancer and lung cancer in females. See supplementary Fig. S4 for more details.

Lung, prostate, colorectal, and stomach cancers in males in Kazakhstan and Uzbekistan had a high incidence among the Central Asian countries, while a small difference in the incidence and mortality of cancers was observed in females; cancers of the breast, cervix uteri, colorectum, corpus uteri, and stomach were the main ones (supplementary Fig. S5). Among the member states of the CIS countries, the highest cancer incidence among both sexes was observed in Belarus and Russia, with the top five most common cancers being those of the prostate, breast, colorectum, lung, and corpus uteri. Similarly, the highest cancer mortality occurred in Armenia and the Republic of Moldova, primarily in association with cancers of the lung, breast, prostate, colorectum, and stomach (supplementary Fig. S6).

The incidence of cancer in the member states of Central and Eastern Europe was the highest among all the member states of the “B&R”; the combined cancer incidence was over ASR 200.0 per 100,000. The cancer mortality rate was relatively low, with an ASR of 100.0 per 100,000 on average. Hungary had the most serious cancer incidence and death rates, with a total incidence rate of ASR 321.1 per 100,000 and a total mortality rate of ASR 125.2 per 100,000. Prostate and breast cancers were the two most commonly diagnosed cancer types, and lung, colorectum, breast, and prostate cancers were the leading causes of cancer death (supplementary Fig. S7).

### Distribution of major cancers in the "B&R" member states

**Fig 2:**
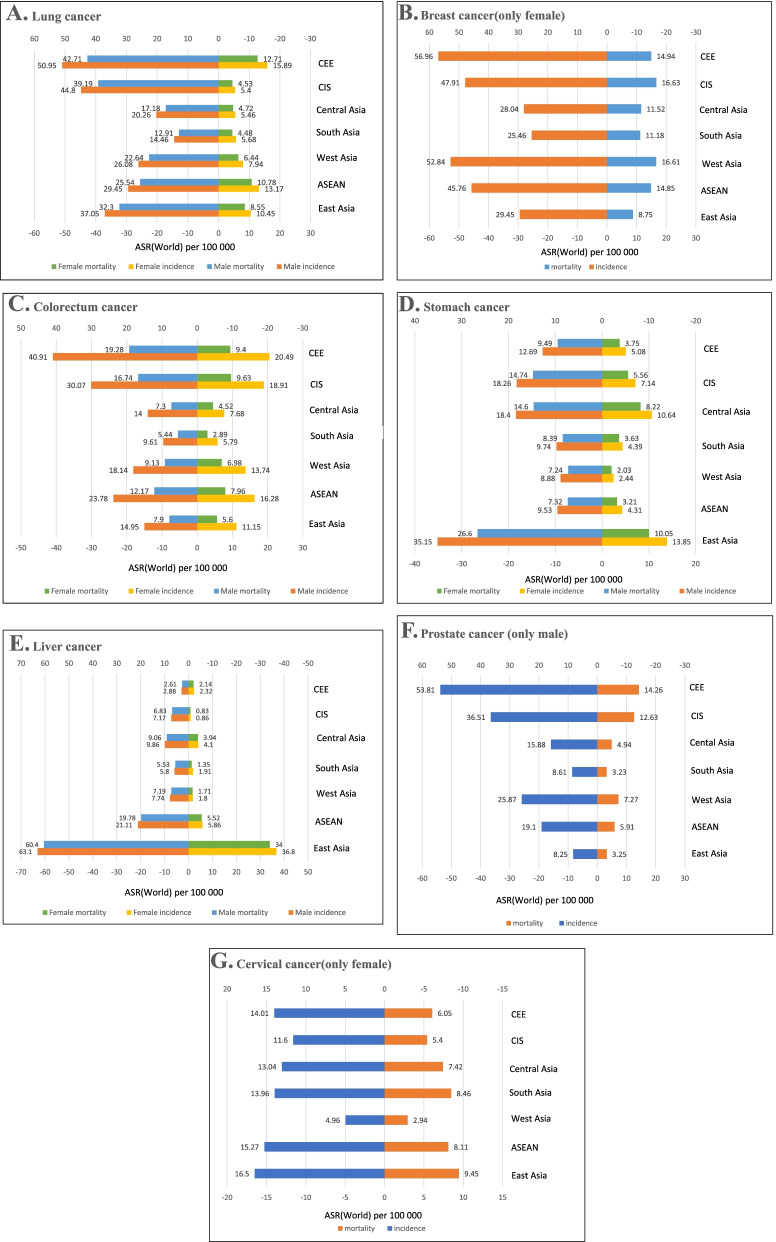
Bar chart of region-specific incidence and mortality ASR by sex for cancers in 2020

The main cancer burdens in the member states of the “B&R” were lung, breast, colorectal, stomach, liver, prostate, and cervical cancer. In terms of the incidence and mortality of lung cancer, CEE member countries carried the heaviest one, and the burden of males was approximately four- to fivefold higher than that of females (Fig. 2A). The incidence of breast cancer showed a greatly difference, ranging from ASR 25.46 per 100,000 to ASR 56.96 per 100,000. However, this was less marked in terms of mortality (Fig. 2B). The burden of colorectal cancer in males was twice that in females, and the burden of incidence was twice that of mortality. The heaviest burden of incidence and mortality of colorectal cancer was observed in CEE member states, followed by the CIS and ASEAN countries (Fig. 2C). East Asian member states had the largest burden of incidence and mortality of stomach cancer, at two to four times and one to five times the rates of other member states, respectively (Fig. 2D). The same was true of liver cancer, which were three to 30 times and six to 30 times the rates that of other member states, respectively (Fig. 2E). The incidence of prostate cancer was relatively high, while the opposite was true of the mortality rate; and the incidence and mortality burden of prostate cancer in CEE member states was the heaviest, with ASR 53.81 per 100,000 and ASR 14.26 per 100,000, respectively (Fig. 2F). The incidence and mortality of cervical cancer were evenly distributed in the member states, with the exception of the lowest incidence in West Asia. The incidence and mortality in other member countries ranged from ASR 11.00 per 100,000 to ASR 17.00 per 100,000 and ASR 5.00 per 100,000 to ASR 10.00 per 100,000 (Fig. 2G).

### Three main risk factors for cancers in the “B&R” member states

**Fig 3:**
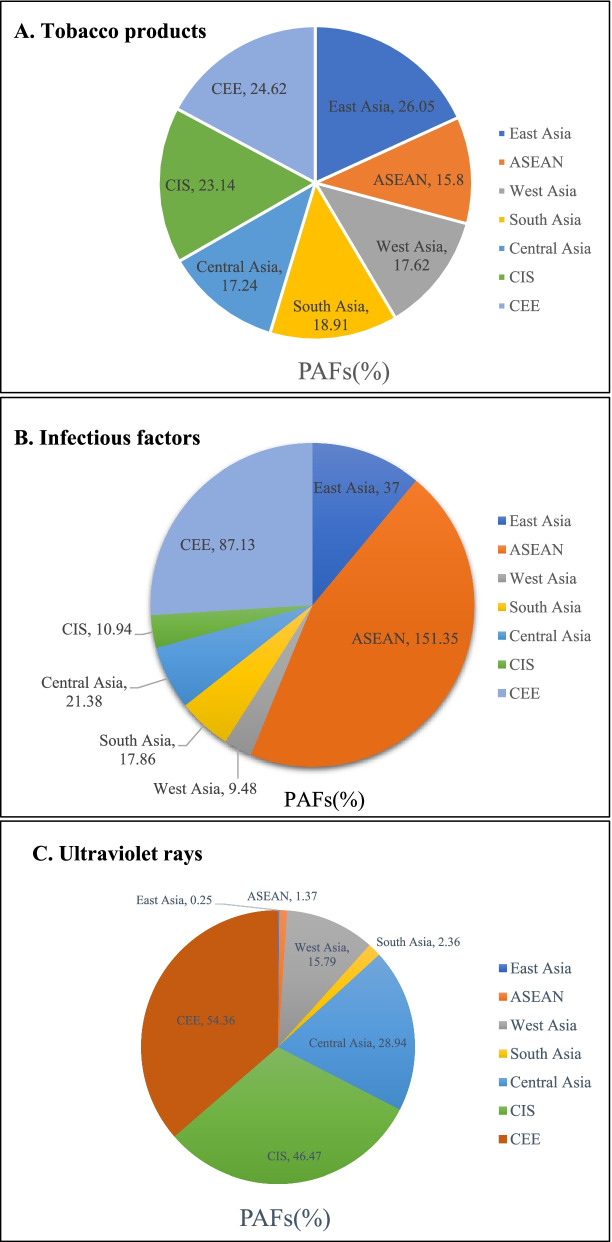
Pie chart of Distribution of major cancer risk factors in the “Belt and Road” geographical location

According to our statistics, tobacco products, infectious factors, and ultraviolet rays were the three main risk factors for cancers in the “B&R” member countries. Tobacco products were the biggest challenge facing all member states, causing the highest burden of cancer incidence and mortality in East Asia and CEE (Fig. 3A). The mean PAF scores of tobacco products were estimated at 30% and above in the following 10 member states: Serbia, Greece, Hungary, Bosnia and Herzegovina, Bulgaria, Croatia, Poland, Romania, Turkey, and China. PAF score above 20% was estimated in a further 27 members. Infectious factors were the most predominant risk factors for cancers in ASEAN, CEE, and East Asia, with PAFs of 151.35%, 87.13%, and 37.00%, respectively (Fig. 3B). There were five member states with more than 30% of PAFs, Vietnam, Singapore, Romania, Mongolia, and Laos, and 11 member states with more than 20%. The cancer burden caused by ultraviolet rays was mainly concentrated in the member states of CEE, CIS, Central Asia, and West Asia (Fig. 3C). Similarly, more than 50% of PAFs, dominated by CEE, were estimated in 18 member states: Estonia, Georgia, Kazakhstan, Latvia, Hungary, Israel, Belarus, Bulgaria, Croatia, Cyprus, Czech Republic, Lithuania, Poland, Romania, Russia, Serbia, Slovenia, Ukraine. There were eight other member states with PAFs above 30%.

## Discussion

In the present study, we analyzed the incidence, mortality, and risk factors of cancer in the member states of the “B&R.” The results show that the main cancer burdens in the member states of the “B&R” were lung, breast, colorectal, stomach, liver, prostate, and cervical cancer. Different member states faced different types of cancer threats to varying degrees. Furthermore, tobacco products, infectious factors, and ultraviolet rays were found to be the three main risk factors of cancer in the “B&R” countries.

Cancer poses a growing global threat to human health [13]. In 134 out of 183 countries, cancer is the first or second leading cause of premature death (i.e., 30–69 years of age), and in another 45 countries, cancer is ranked third or fourth [14]. Death rates from NCDs, particularly cancer, are declining in most high-income countries, but not in low-income countries. By 2040, cancer incidence is projected to increase by the largest percentage in countries with low and medium HDI. From 2018 to 2040, the estimated increase is 100% in the low HDI tier and 75% in the middle HDI tier, looking at population changes alone [3]. Most of countries of the “B&R” are developing countries, other than Cyprus, Czech Republic, Greece, Hungary, Israel, and Singapore. Lung cancer, breast cancer, gastrointestinal cancer, prostate cancer, cervical cancer, thyroid cancer, and uterine cancer are common challenges that the "B&R" countries must face together. Countries with low to medium HDI levels are currently ill-equipped to cope with the coming increase in the cancer burden. Thus, the health "One Belt One Road" initiative offers new solutions to the challenge of the cancer burden in –low and medium HDI countries. Strengthening regional scientific and technological cooperation through targeted, resource-sharing, effective, and cost-effective means, such as establishing combined laboratories or research centers and transferring international technology and knowledge, are necessary measures to reduce the burden of cancers [15, 16]. Furthermore, countries with high MIR often lack proper health policies or recommendations for cancer control. A multipronged approach is required to strengthen the health care infrastructure in these countries, which includes collaboration between national governments, on-the-ground primary care providers, medical subspecialists, and the wider public health community.

Cancer is a complex disease, and the incidence and death of cancer vary widely among member states. In this study, the highest lung and digestive tract cancer incidence occurred in East Asia, whereas breast cancer and lung cancer showed predominance in ASEAN, West Asia, South Asia, and Central Asia. In South Asia, lip cancer and oral cavity cancer were the leading causes of cancer deaths. South and Central Asia accounted for more than a third of the global oral cancer burden. In 2018, India had the highest burden of oral cancer, with approximately 120,000 new cases [3]. In member countries in CIS and CEE, colorectal cancer and lung cancer were the most diagnosed cancers with the highest mortality, of which the CEE countries had a high incidence of predominantly male cancers, such as bladder and prostate cancer. These variations might be related to disparities in genetic vulnerability, lifestyle, and local environmental exposures among the different locations. For example, the ASR of alcohol-induced cancer deaths and DALY varied greatly between countries and regions. The highest burden of alcohol-induced cancers was observed in Eastern Europe, and the lowest was observed in North Africa and the Middle East [17]. In countries with a high HDI, the risk of colorectal cancer tends to be relatively high, and incidence and mortality rates have stabilized or declined [18]. The increased incidence of obesity and the increased consumption of dairy products and calcium have previously been associated with an increase in the risk of prostate cancer. In the black population globally, for instance, the incidence of prostate cancer is much higher [19]. Further, the inherent and growing disparities in medical practice and health infrastructure within and between countries also affect the patterns and trends in cancer mortality [20–23]. Two major factors in the decline in prostate cancer deaths are likely to be PSA testing (i.e., more cancers are found at an earlier stage) and better management of patients [24].

Lung cancer remains the most common type of cancer globally, with an estimated 2.1 million new cases and 1.8 million deaths worldwide in 2018 [3]. Lung cancer is the leading cause of cancer incidence and mortality in males among the “B&R” countries as well. Smoking is the leading cause of lung cancer, accounting for 63% of all lung cancer deaths globally and more than 90% of deaths in countries where smoking is common in both sexes [25]. However, the burden of cancer deaths from tobacco products in the “B&R” member countries is heavy, and tobacco control policies have failed to be adequately implemented. In 2018, Turkey, the only member country, implemented five of the six MPOWER measures. Ten countries (Bulgaria, the Czech Republic, Egypt, Nepal, Russia, Saudi Arabia, Slovenia, Thailand, Greece, and Iran) implemented three of the six MPOWER measures, but seven countries have not implemented the MPOWER measures. Furthermore, emerging tobacco products have challenged regulatory approaches to control the public health burden of tobacco-related cancers. Further research should be conducted to determine the risks of emerging tobacco products to accelerate progress in tobacco control while limiting the hazards of existing traditional tobacco products.

The cancer burden among females in the “B&R” countries is considerably high. Breast cancer remains a serious cancer burden in women. According to the latest data in 2020, breast cancer surpassed lung cancer, and for the first time, became the most common cancer in the world (accounting for 11.7% of new cases), ranking the first among the types of cancer worldwide [26]. The rising incidence of breast cancer is associated with the trend of an earlier age at menarche, later age at birth, and parity [27]. Therefore, the incidence of breast cancer can be reduced through regular physical examination screening, postponement or reduction of fertility, reasonable weight control, moderate increases in the amount of exercise, and other control measures. However, nine of the “B&R” countries (Afghanistan, Azerbaijan, Brunei, Cambodia, Nepal, Tajikistan, Uzbekistan, Yemen, and Pakistan) do not yet conduct breast cancer screening. The starting age of the target population for breast cancer screening is mainly between 30 and 50 years of age, and the age of screening in Malaysia, the Maldives, and Bhutan is below 20 years of age. Developing scientific and effective screening methods for breast cancer, improving breast cancer screening programs, and actively carrying out early diagnosis and treatment will remain the key points of breast cancer prevention and treatment in the future.

In addition, cervical cancer carries the highest burden in less developed countries and regions in the “B&R” countries [28], and nearly 100% of cervical cancers are caused by high-risk human papilloma virus (HPV) infection [29]. While cervical cancer is the only common cancer with a clear etiology and can be completely effectively prevented by tertiary prevention [30], and HPV vaccination can protect at least 80% of the target population [31], only 18 of the member countries have HPV vaccination programs in place, of which, vaccination coverage of Bhutan, Brunei, Malaysia, and Turkey is more than 80%, while that in Armenia, the Philippines, Singapore, and Indonesia is less than 5%. Moreover, 12 member states (i.e., Afghan, Egypt, Oman, Saudi Arabia, Tajikistan, Uzbekistan, Yemen, Pakistan, Kyrgyzstan, Kuwait, Iraq, and Iraq) do not yet carry out cervical cancer screening. Therefore, the implementation of an HPV vaccination plan and the development of the corresponding screening strategy is the main task of cervical cancer prevention and control in the “B&R” member states.

Infectious agents are the key cause of cancer, particularly in low- and middle-income countries. It was estimated that in 2018, about one-eighth of the 18 million new cancer cases in the world were caused by infection [32]. Hepatitis B virus (HBV) infection, Hepatitis C virus (HCV) infection, liver fluke, and Clonorchis’s sinensis remain the leading infectious factors leading to cancer in low- and middle-income countries [33, 34]. Among other infectious factors, HTLV-1 and large parasites contribute little to the global burden of cancer but are a significant cause of cancer in endemic populations [35]. Moreover, Helicobacter pylori is responsible for a large number of gastrointestinal cancers and deaths [36]. Combination therapy with antibacterial drugs has potential preventive effects. Since 1982, vaccines have been available to prevent HBV, and direct-acting antiviral drugs have the potential to cure more than 95% of people infected with HCV [3]. Adequate infection control strategies, including cheap and reliable point-of-care diagnostic tests for specific infectious agents for screening, effective treatments, and therapeutic and preventive vaccines, should all play a broader role in cancer control programs. However, to realize these aspirations, a large amount of international investment is required. Therefore, the "B&R" initiative provides a new platform for international health cooperation.

Ultraviolet radiation directly or indirectly induces DNA damage, leads to mutations, triggers inflammation and immunosuppression, and finally leads to tumor growth. Photocarcinogenesis is a complex, multi-step pathway, which is triggered by the formation of dipyrimidine photoproducts and leads to the formation of mutations (initial stage). Sunburn and inflammation caused by persistent DNA lesions (including dipyrimidine photoproducts and oxidative DNA lesions) play a role in promoting the process of photocarcinogenesis [37]. The dipyrimidine photoproduct triggers ultraviolet-induced immunosuppression, leading to the failure of immune surveillance and the growth and development of cancer cells [38]. The incidence of both melanoma and non-melanoma skin cancer is increasing worldwide, not only in Caucasians [39], but also in Asians, and UV mutations are significantly more common in sun-exposed skin areas than in non-sun-exposed skin areas [40]. The most effective way to reduce the incidence of skin cancer is to avoid unnecessary sun exposure and take personal precautions, such as wearing protective clothing and hats, applying sunscreen, and staying in a cool place, which can significantly reduce the risk of sun damage.

In the wake of the COVID-19 pandemic, awareness of global public health has become clearer, and policy has played a crucial role in promoting global public health. By studying and comparing the incidence, mortality and risk factors of cancer in the “B&R” countries, this study identified different cancer challenges faced by different countries, proposed cancer prevention and control measures applicable to each country, and provided suggestions for health cooperation programs in “B&R” countries. However, the data sources involved in this study are extensive, and the data quality will be uneven. Readers are suggested to quote and extrapolate after reading the analysis methods and data sources of this study in detail.

## Conclusion

In summary, the challenges posed by cancer are consistent in the “B&R” member countries. Lung cancer, breast cancer, gastrointestinal cancer, prostate cancer, cervical cancer, thyroid cancer, and uterine cancer were found to be the biggest cancer burdens in the “B&R” countries, and tobacco products, infectious factors, and ultraviolet rays were the main risk factors. Moreover, the corresponding cancer prevention and control policies need to be improved. Therefore, in the context of the health "One Belt One Road" initiative, the collaboration between multiple stakeholders and the sharing of resources will play a positive role in jointly coping with the risks and challenges brought by cancer and promoting the positive development of the health and medical undertaking of all member states and the world at large.

## Statement

All methods were carried out in accordance with relevant guidelines and regulations.

## Supplementary Information

**Additional File 1. Supplementary file 1** Comparison of estimated 2020 ASR (world) among East Asia countries, all ages.

**Additional File 2. Supplementary file 2 **Comparison of estimated 2020 ASR (world) among ASEAN countries, all ages.

**Additional File 3. Supplementary file 3** Comparison of estimated 2020 ASR (world) among West Asia countries, all ages.

**Additional File 4. Supplementary file 4** Comparison of estimated 2020 ASR (world) among South Asia countries, all ages.

**Additional File 5. Supplementary file 5** Comparison of estimated 2020 ASR (world) among Central Asia countries, all ages.

**Additional File 6. Supplementary file 6** Comparison of estimated 2020 ASR (world) among CIS countries, all ages.

**Additional File 7. Supplementary file 7** Comparison of estimated 2020 ASR (world) among Central and eastern Europe countries, all ages.

## Data Availability

The data that support the findings of this study are available via the Global Cancer Observatory (https://gco.iarc.fr/) and Cancer Country Profiles (https://www.who.int/countries).

## Abbreviations

HDI: Human Development Index
CMNNDS: Communicable, Maternal, Neonatal, and Nutritional diseases
NCDs: Non-Communicable Diseases
PBCRs: Population-Based Cancer Registries
ASRs: Age-Standardized Rates
MIR: The mortality­to­incidence ratio
PAFs: Population Attributable Fractions
ASEAN: The Association of South-East Asian Nations
CIS: The Commonwealth of Independent States
CEE: Central and Eastern Europe
HPV: Human Papilloma Virus
HBV: Hepatitis B Virus
HCV: Hepatitis C Virus
