# Supplementary material for: Cancer incidence and mortality and risk factors in member countries of the " Belt and Road " initiative
Source: BMC Cancer. 2022 May 25;22:582. doi: 10.1186/s12885-022-09657-3 (PMC9132358; doi:10.1186/s12885-022-09657-3)
Supplement: Supplementary file 3 — Additional File 3. Supplementary file 3 Comparison of estimated 2020 ASR (world) among West Asia countries, all ages. [file 12885_2022_9657_MOESM3_ESM.docx]

Fig. S3. Comparison of estimated 2020 ASR (world) among West Asia countries, all ages.
